# Michigan State Board of Health

**Published:** 1884-03

**Authors:** 


THE MICHIGAN STATE BOARD OF HEALTH.

The regular quarterly meeting of the Michigan State Board of Health was held in the office of the Board at Lansing, Michigan, January 8, 1884, the following members being present: Jno. Avery, M.D., of Greenville, President; J. H. Kellogg, M.D., of Battle Creek; Victor C. Vaughan, M.D., of Ann Arbor; C. V. Tyler, M.D., of Bay City; and Henry B. Baker, M.D., Secretary.

The recommendations to the Warden of the State House of Correction, by Dr. Jno. Avery, Committee on Buildings, Ventilation, etc., under date of September 7, 1883, for improving the ventilation of the shops at the State House of Correction, at Ionia, were ordered printed in the next Annual Report.

The Secretary read a resumé of recent work of other boards of health; also a summary of the work of the office of this Board during the last quarter, which showed that a successful sanitary convention had been held at Ionia; that partial arrangements had been made for holding one at Hillsdale; that the proceedings of the Muskegon, Pontiac, and Bay City Conventions had been printed; that special meetings of the Board had been called at the State Reform School to examine plans for a new building; at Detroit to attend the American Public Health Association; and at Ionia at the time of the Convention; that a leaflet on contagious diseases had been translated into French, Danish-Norwegian, and Swedish, for general distribution among citizens of Michigan, who speak those languages; that a very general distribution of circulars on communicable diseases and on the work of health officers had been made to the health officers of cities, villages and townships in Michigan; that similar documents were distributed at Ionia, and at the State Teachers' Association; that circulars, blanks, etc., had been sent to health officers and clerks of all cities, villages and townships in Michigan for their annual report, including that of the diseases dangerous to public health; that notice had been sent to health authorities in several parts of the State warning of the shipment of diseased cattle into such localities; that the regular distribution of weekly bulletins of sickness and of meteorology, the yearly distribution of material for meteorological reports, and the quarterly distribution of blanks to observers of diseases had been made.

The Secretary presented reports from Dr. Hazlewood, who as committee of the Board attended the Sanitary Convention at London, Ontario, and the meeting of the Board of Corrections and Charities at East Saginaw. In connection with the first report, Dr. Hazlewood described the water supply of that city (London), and the Secretary, who also attended the Convention, described a visit to the Asylum of the Insane near London, Ontario.

By request of the State Board of Corrections and Charities, committees were appointed to examine and report on the sanitary condition of the jails, asylums, etc., in several counties in Michigan.

Drs. Avery and Kellogg were appointed a committee to investigate and report at next regular meeting on the sewerage, ventilation, etc., of the Capitol building.

A committee, consisting of Drs. Baker, Kellogg and Vaughan, were appointed to examine and report on the sanitary condition and needs of the State Reform School and surroundings.

Dr. Kellogg presented and read portions of a very interesting report on the present knowledge respecting diphtheria, which will be published in the next Annual Report.

Considerable discussion occurred over the examination of text books on physiology and hygiene with reference to alcohol and other narcotics. Only four books had been presented for examination. The committee reported relative to these books, and asked to be discharged; but the committee was continued, Prof. Vaughan added to it; it was directed to confer with a similar committee from the State Board of Education, and it is to report again at the next regular meeting of the Board, April 8. It is hoped that publishers of school-books will give early attention to this subject, and that more than one book can be approved at that time.

